# Linking karyotypes with DNA barcodes: proposal for a new standard in chromosomal analysis with an example based on the study of Neotropical Nymphalidae (Lepidoptera)

**DOI:** 10.3897/CompCytogen.v13i4.48368

**Published:** 2019-12-17

**Authors:** Vladimir A. Lukhtanov, Yaroslavna Iashenkova

**Affiliations:** 1 Department of Karyosystematics, Zoological Institute of the Russian Academy of Sciences, Universitetskaya emb. 1, St. Petersburg 199034, Russia; 2 Department of Entomology, St. Petersburg State University, Universitetskaya emb. 7/9, St. Petersburg 199034, Russia; 3 Department of Genetics and Biotechnology, St. Petersburg State University, Universitetskaya emb. 7/9, St. Petersburg 199034, Russia

**Keywords:** karyotype, DNA barcoding, *COI*, meiosis, metaphase, Lepidoptera, Nymphalidae, Biblidinae, Danainae, Ithomiini, Peru

## Abstract

Chromosomal data are important for taxonomists, cytogeneticists and evolutionary biologists; however, the value of these data decreases sharply if they are obtained for individuals with inaccurate species identification or unclear species identity. To avoid this problem, here we suggest linking each karyotyped sample with its DNA barcode, photograph and precise geographic data, providing an opportunity for unambiguous identification of described taxa and for delimitation of undescribed species. Using this approach, we present new data on chromosome number diversity in neotropical butterflies of the subfamily Biblidinae (genus *Vila* Kirby, 1871) and the tribe Ithomiini (genera *Oleria* Hübner, 1816, *Ithomia* Hübner, 1816, *Godyris* Boisduval, 1870, *Hypothyris* Hübner, 1821, *Napeogenes* Bates, 1862, *Pseudoscada* Godman et Salvin, 1879 and *Hyposcada* Godman et Salvin, 1879). Combining new and previously published data we show that the species complex *Oleria onega* (Hewitson, [1852]) includes three discrete chromosomal clusters (with haploid chromosome numbers n = 15, n = 22 and n = 30) and at least four DNA barcode clusters. Then we discuss how the incomplete connection between these chromosomal and molecular data (karyotypes and DNA barcodes were obtained for different sets of individuals) complicates the taxonomic interpretation of the discovered clusters.

## Introduction

Chromosomal data are an important source of information for taxonomic, evolutionary and comparative phylogenetic studies (White 1973). However, the application of these data is often difficult because of unclear taxonomic identity (e.g. Petrova et al. 2015) or doubtful species identification (or even due to the lack of a species identification) of the samples that were used as vouchers for karyotype analysis [e.g. some samples and identifications in Robinson (1971) and Brown et al. (2004)]. Theoretically, one can try to find these samples, provided that they were neatly labeled and can be recognized, are stored in accessible museums and have not been lost, and then check their identification using taxonomic literature or comparison with type specimens. However, it is complicated and almost impossible in practice.

To avoid this problem, here we suggest linking each karyotyped sample with its DNA barcode. It was empirically demonstrated that the mitochondrial DNA barcode, a relatively short fragment of the mitochondrial *COI* gene (658 base pairs) (i.e., a negligible part of the genome in terms of size), could differentiate up to 95% of species in many taxa (Hebert et al. 2003, 2004; Hebert and Gregory 2005; Hajibabaei et al. 2006; Lukhtanov et al. 2009). In addition, the barcoding DNA protocol provides a standardized system for storing information on vouchers that served as the basis for DNA barcoding, including the image, the exact label and the storage location of the samples. This makes it possible, if necessary, to relatively easily find and re-examine a voucher.

Obtaining barcodes is currently a simple technical task, which can be carried out in almost any laboratory or on a commercial basis. Our personal experience, based on a molecular analysis of the fauna of Central Asia, Eastern Europe and Western Asia (Lukhtanov et al. 2009, 2016; Lukhtanov 2017; Pazhenkova and Lukhtanov 2019), shows that if there are barcode libraries (Ward et al. 2009; Dincă et al. 2011) for a given region and for a given taxonomic group, barcodes ensure almost 100% success of species identification. Even if such a library is not currently available for a group or region under study, the presence of a barcode makes it possible to reliably identify the sample in the future. Thus, linking karyotypes with DNA barcodes resolves the problem of reliable species identification.

Additionally, combination of DNA barcodes and karyotypes represents a powerful tool for detection, delimitation and description of unrecognized species (Lukhtanov et al. 2015; Vishnevskaya et al. 2016, 2018). Therefore, linking karyotypes with DNA barcodes, potentially resolves the problem of unclear species identity in chromosomal studies.

The approach based on combination of chromosomal and DNA barcode data has been already used in different studies on butterflies (Lukhtanov et al. 2014, 2015; Lukhtanov and Dantchenko 2017), fish (Marques et al. 2013), lizards (de Matos et al. 2016), mammals (Tavares et al. 2015) and mussels (Garcia-Souto et al. 2017). However, its principles have not been explicitly formulated.

In this paper, we demonstrate the algorithm, features and capabilities of the proposed approach with the butterflies of the Neotropical fauna.

## Material and methods

### Samples

The samples were collected in Peru in 2013 by V.A.Lukhtanov. The information on localities where the specimens were collected is presented in the Table 1. The morphology-based species identification was carried out by comparing the specimens with butterfly images figured at Butterflies of America site (https://www.butterfliesofamerica.com/L/Nymphalidae.htm). Photographs of all specimens used in the analysis, as well as collecting data, are available on the Barcode of Life Data System (BOLD) at http://www.boldsystems.org/. The specimens are deposited in the Zoological Institute of the Russian Academy of Sciences, St. Petersburg, Russia.

**Table 1.** List of the samples of the genera *Oleria* Hübner, 1816, *Ithomia* Hübner, 1816, *Vila* Kirby, 1871, *Pseudoscada* Godman et Salvin, 1879, *Godyris* Boisduval, 1870, *Hypothyris* Hübner, 1821, *Napeogenes* Bates, 1862 and *Hyposcada* Godman et Salvin, 1879 collected by V.A.Lukhtanov and used in the study.

**Id**	**BOLD Id**	**Genus**	**Species**	**N**	**Exact site**	**Latitude / Longitude**	**Altitude**	**Collection date**
A107	NOB001-17	* Oleria *	* didymaea ramona *	n = 22	60 km SSW Ikitos, Puente Itaya	04°11'47"S, 73°28'39"W	114 m	30 August 2013
A108	NOB002-17	* Ithomia *	* salapia *	n = 34	60 km SSW Ikitos, Puente Itaya	04°11'47"S, 73°28'39"W	114 m	30 August 2013
A111	NOB004-17	*Vila*	*emilia*	n = 30	60 km SSW Ikitos, Puente Itaya	04°11'47"S, 73°28'39"W	114 m	30 August 2013
A112	NOB005-17	*Vila*	*emilia*	–	60 km SSW Ikitos, Puente Itaya	04°11'47"S, 73°28'39"W	114 m	30 August 2013
A113	NOB006-17	*Vila*	*emilia*	n = 30	60 km SSW Ikitos, Puente Itaya	04°11'47"S, 73°28'39"W	114 m	30 August 2013
A115	NOB007-17	*Vila*	*emilia*	n = 30	60 km SSW Ikitos, Puente Itaya	04°11'47"S, 73°28'39"W	114 m	30 August 2013
A121	NOB008-17	* Oleria *	* gunilla serdolis *	n = 11	Tingo Maria	09°21'02"S, 76°03'21"W	835 m	4 September 2013
A122	NOB009-17	* Oleria *	* gunilla serdolis *	n = 11	Tingo Maria	09°21'02"S, 76°03'21"W	835 m	4 September 2013
A123	NOB010-17	* Oleria *	*gunillaserdolis*)	n = 11	Tingo Maria	09°21'02"S, 76°03'21"W	835 m	4 September 2013
A125	NOB011-17	* Oleria *	* onega *	n = 15	Tingo Maria	09°21'02"S, 76°03'21"W	835 m	4 September 2013
A127	NOB012-17	* Oleria *	* gunilla serdolis *	n = 11	Tingo Maria	09°21'02"S, 76°03'21"W	835 m	4 September 2013
A124	NOB013-17	* Oleria *	* onega *	n = 15	Tingo Maria	09°21'02"S, 76°03'21"W	835 m	4 September 2013
A129	n/a	* Pseudoscada *	* timna *	n = 15	Tingo Maria	09°21'02"S, 76°03'21"W	835 m	4 September 2013
A130	NOB014-17	* Ithomia *	* salapia *	n = 34	Tingo Maria	09°21'02"S, 76°03'21"W	835 m	4 September 2013
A131	NOB015-17	* Godyris *	* zavaleta *	n = 33,35	Tingo Maria	09°21'02"S, 76°03'21"W	835 m	4 September 2013
A132	NOB016-17	* Ithomia *	* salapia *	n = 35	Tingo Maria	09°21'02"S, 76°03'21"W	835 m	4 September 2013
A133	NOB017-17	* Ithomia *	* salapia *	n = 36	Tingo Maria	09°21'02"S, 76°03'21"W	835 m	4 September 2013
A135	NOB018-17	* Ithomia *	* salapia *	n = 36	Tingo Maria	09°21'02"S, 76°03'21"W	835 m	4 September 2013
A136	NOB019-17	* Hypothyris *	* euclea *	n = 14	Tingo Maria	09°21'02"S, 76°03'21"W	835 m	4 September 2013
A137	NOB020-17	* Napeogenes *	* sylphis *	n = 14	Tingo Maria	09°21'02"S, 76°03'21"W	835 m	4 September 2013
A140	NOB021-17	* Hyposcada *	* kena *	n = 14	Cayumba	09°29'25"S, 75°56'46"W	1020 m	5 September 2013
A141	NOB022-17	* Oleria *	* onega *	n = 15	Cayumba	09°29'25"S, 75°56'46"W	1020 m	5 September 2013
A142	NOB023-17	* Oleria *	* onega *	n = 15	Cayumba	09°29'25"S, 75°56'46"W	1020 m	5 September 2013
A143	NOB024-17	* Oleria *	* onega *	n = 15	Cayumba	09°29'25"S, 75°56'46"W	1020 m	5 September 2013
A144	NOB025-17	* Oleria *	* onega *	n = 15	Cayumba	09°29'25"S, 75°56'46"W	1020 m	5 September 2013
A145	NOB026-17	* Godyris *	* dircenna *	n = 36	Cayumba	09°29'43"S, 75°58'01"W	786 m	6 September 2013

### Chromosomal analysis

**Figures 1–19.**
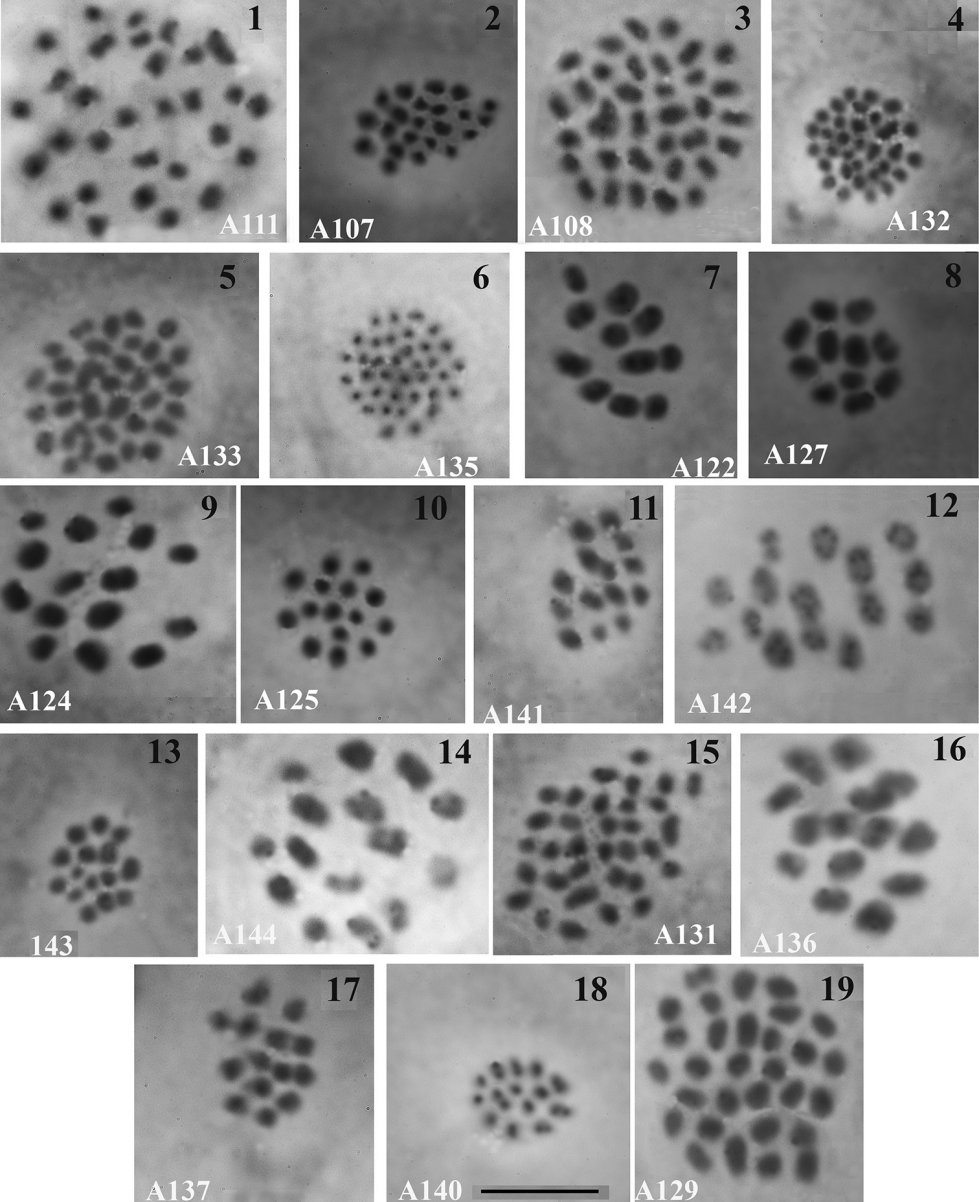
Male metaphase I (MI) and II (MII) plates of Ithomiini and Biblidinae**1** A111, *Vila emilia*, MI, n = 30 **2** A107, *Oleria didymaea ramona*, MI, n = 22 **3** A108, *Ithomia salapia*, MI, n = 34 **4** A132, *Ithomia salapia*, MII, n = 35 **5** A133, *Ithomia salapia*, MI, n = 36 **6** A135, *Ithomia salapia*, MII, n = 36 **7 8** A122 *Oleria gunilla serdolis*, MI, n = 11 **9** A124, *Oleria onega*, MI, n = 15 **10** A125, *Oleria onega*, MII, n = 15 **11** A141, *Oleria onega*, MI, n = 15 **12** A142, *Oleria onega*, MI, n = 15 **13** A143, *Oleria onega*, MII, n = 15 **14** A144, *Oleria onega*, MI, n = 15 **15** A131, *Godyris zavaleta*, MI, n = 33 **16** A136, *Hypothyris euclea*, MI, n = 14 **17** A137, *Napeogenes sylphis*, MI, n = 14 **18** A140, *Hyposcada kena*, MII, n = 14 **19** A129, *Pseudoscada timna*, MI, n = 30. Scale bar: 10 μ in all figures.

Gonads were removed from the abdomen and placed into freshly prepared fixative (3:1; 96% ethanol and glacial acetic acid) directly after capturing the butterfly in the field. Testes were stored in the fixative for 3–36 months at +4 °C. Then the gonads were stained in 2% acetic orcein for 30–60 days at +18–20 °C. Karyotypes (Figs 1–19) were analyzed as previously described (Przybyłowicz et al. 2014; Lukhtanov and Shapoval 2017). Briefly, the stained testes were placed in a drop of 40% lactic acid on a slide, and spermatocysts were dissected from gonad membranes using entomological pins before covering everything with a coverslip. Different degrees of chromosome spreading were observed by gradually increasing the pressure on the coverslip. Haploid chromosome numbers (n) were counted at meiotic metaphase I (MI) and metaphase II (MII).

### DNA barcoding

Standard *COI* barcodes (658-bp 5’ segment of mitochondrial cytochrome oxidase subunit I) were studied. Legs were sampled from the karyotyped specimens, and sequence data from the DNA barcode region of *COI* were obtained at the Canadian Centre for DNA Barcoding (CCDB, Biodiversity Institute of Ontario, University of Guelph) using primers and protocols described in Hajibabaei et al. (2005), Ivanova et al. (2006) and deWaard et al. (2008).

The DNA-barcode-based species identification was carried out by using the BOLDSYSTEMS Identification Engine (http://www.boldsystems.org/index.php/IDS_OpenIdEngine).

**Figure 20.**
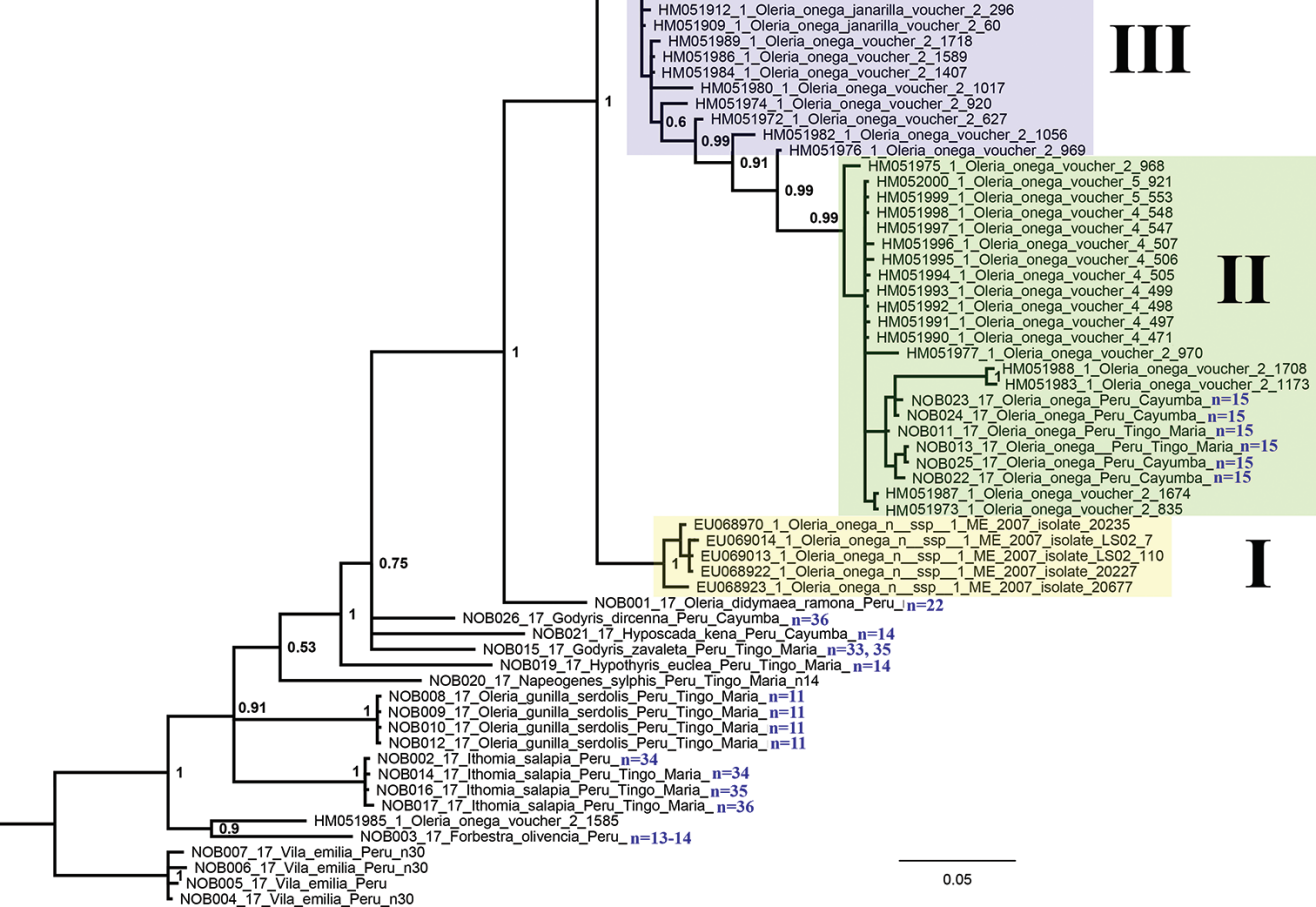
Fragment of the Bayesian majority rule consensus tree of the analyzed samples of Ithomiini inferred from *COI* sequences. I, II and III are the recovered clusters of the *Oleria onega* species complex (see Fig. 21 for the complete structure of the cluster III and the cluster IV). Haploid chromosome numbers (n) are shown after the tip labels. *Vila emilia* (subfamily Biblidinae) was used to root the tree. Bayesian posterior probabilities higher than 0.5 are shown next to the recovered branches.

**Figure 21.**
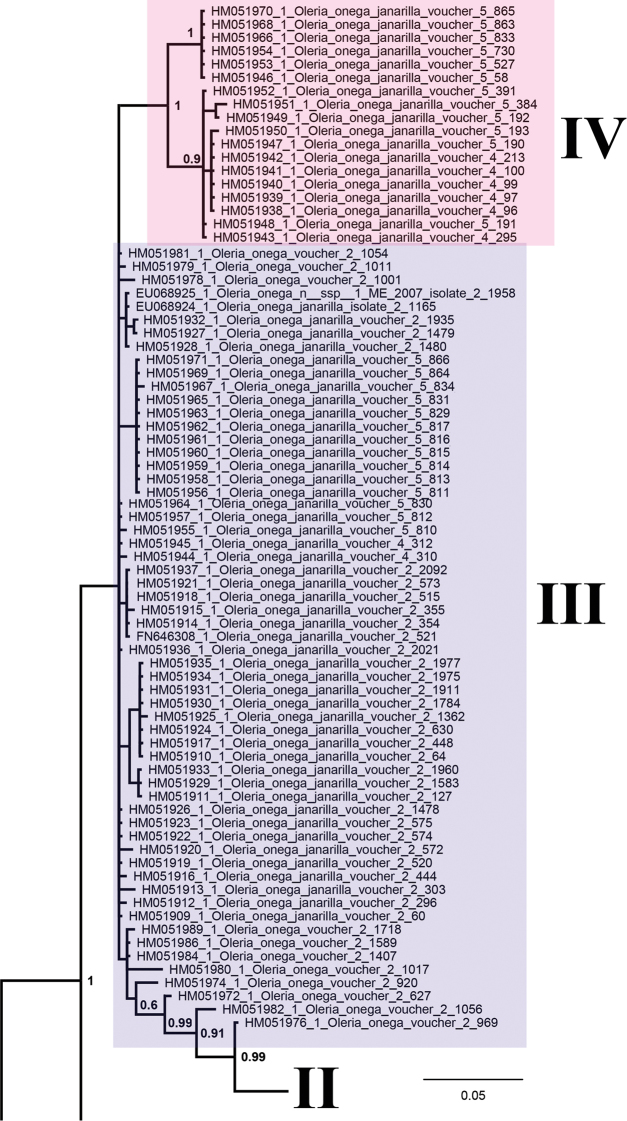
Fragment of the Bayesian majority rule consensus tree of the analyzed samples of Ithomiini inferred from *COI* sequences. The clusters III and IV of the *Oleria onega* species complex are shown. Bayesian posterior probabilities higher than 0.5 are shown next to the recovered branches.

The Bayesian majority rule consensus tree of the analyzed samples (Figs 20, 21) was constructed as previously described (Sahoo et al. 2016; Lukhtanov 2017; Lukhtanov and Dantchenko 2017) using the sequences obtained in this study as well as the published sequences uploaded from GenBank (de-Silva et al. 2010). Briefly, sequences were aligned using the BioEdit software (Hall 1999) and edited manually. The Bayesian analysis was performed using the program MrBayes 3.2 (Ronquist et al. 2012) with default settings as suggested by Mesquite (Maddison and Maddison 2015): burn-in = 0.25, nst = 6 (GTR + I + G). Two runs of 10,000,000 generations with four chains (one cold and three heated) were performed. The consensus of the obtained trees was visualised using FigTree 1.3.1 (http://tree.bio.ed.ac.uk/software/figtree/).

## Results

### Karyotypes

#### Subfamily Biblidinae

***Vila emilia* (Cramer, 1779)**

Fig. 1

The meiotic karyotype was found to include 30 bivalents of similar size.

#### Subfamily Danainae

##### Tribe Ithomiini

***Oleria didymaea ramona* (Haensch, 1909)**

Fig. 2

The meiotic karyotype was found to include 22 bivalents of similar size.

***Ithomia salapia* Hewitson, [1853**]

Figs 3–6

The meiotic karyotype was found to include 34 bivalents in a single studied specimen from Puente Itaya (Peru, 60 km SSW Ikitos). One bivalent was slightly larger than the rest ones. The meiotic karyotype was found to include 35–36 bivalents of similar size in the specimens from Tingo Maria.

***Oleria gunilla serdolis* (Haensch, 1909)**

Figs 7, 8

The meiotic karyotype was found to include 11 bivalents. Two bivalents were larger than the other nine ones.

***Oleria onega* (Hewitson, [1852])**

Figs 9–14

The meiotic karyotype was found to include 15 bivalents. The bivalents had different sizes and shapes.

***Godyris zavaleta* (Hewitson, [1855])**

Fig. 15

The meiotic karyotype was found to include cells with 33 and 35 chromosomal elements, presumably bivalents. 34 bivalents were counted in a single studied specimen from Tingo Maria.

***Hypothyris euclea* (Godart, 1819)**

Fig. 16

The meiotic karyotype was found to include 14 bivalents of similar size.

***Napeogenes sylphis* (Guérin-Méneville, [1844])**

Fig. 17

The meiotic karyotype was found to include 14 bivalents of similar size.

***Hyposcada kena* (Hewitson, 1872)**

Fig. 18

The meiotic karyotype was found to include 14 bivalents. The bivalents had different sizes and shapes.

***Pseudoscada timna* (Hewitson, [1855])**

Fig. 19

The meiotic karyotype was found to include 30 bivalents of similar size. The bivalents formed a gradient size row.

### DNA barcodes

All studied species were found to be significantly differentiated with respect to the DNA barcode region and formed distinct clusters on the BI tree (Fig. 20). However, if additional sequences from GenBank were added, the picture became more intricate. Particularly, *Oleria onega* was found to have very complicated structure with numerous differentiated haplotypes forming three monophyletic and one paraphyletic clusters (Figs 20, 21). The karyotyped samples of this species with the chromosome number n = 15 were found to belong to the cluster II.

## Discussion

The Neotropics is one of the most species-rich regions of the world, and the nymphalids are the most speciose butterfly family (Van Nieukerken et al. 2011). Therefore, it is not surprising that the neotropical fauna of Nymphalidae is very rich in species (site (https://www.butterfliesofamerica.com/L/Nymphalidae.htm).

Chromosomal studies represent only a small part of the Neotropical nymphalid diversity (de Lesse 1967, 1970a, b; de Lesse and Brown 1971; Wesley and Emmel 1975; Suomalainen and Brown 1984; Brown et al. 1992, 2004, 2007a, b; McClure et al. 2017; Lukhtanov 2019a). However, they demonstrate an extremely high level of the interspecific karyotype variation and a potential for solving taxonomic problems within the South American nymphalid species. This potential is practically not used (but see: Suomalainen and Brown 1984; Constantino and Salazar 2010; McClure et al. 2017) in opposite to the numerous chromosomally based taxonomic studies in palearctic butterflies (Lorković 1958; de Lesse 1960; Lukhtanov et al. 2011, 2015; Talavera et al. 2013).

In this study we suggest a plan for further analysis of the Neotropical Nymphalidae based on a parallel analysis of chromosomal and molecular markers.

Using this approach, we confirm the previously published data on the karyotypes of *Godyris dircenna* (n = 36), *Hypothyris euclea* (n = 14), *Napeogenes sylphis* (n = 14) and *Oleria gunilla* (n = 11) (Brown et al. 2004).

Haploid chromosome number n=30 is found by us in *Pseudoscada timna*, whereas n = 31 was reported for this taxon by Brown et al. (2004).

We provide the first data on karyotypes of *Vila emilia* and demonstrate a high interspecific chromosome number variation in this genus (previously n = 15 was reported for an unidentified *Vila* species from western Brazil; Brown et al. 2007a).

We show chromosome number n = 14 for *Hyposcada kena* confirming high level of interspecific variation in the genus *Hyposcada* (from n = 12 to n = 19) (Brown et al. 2004).

Different chromosome numbers were previously reported for *Godyris zavaleta* by Brown et al. (2004): n = 46 (on the page 220–221), n = 35–45 (p. 222), n = 36–46 (p. 224), n = 40 (p. 229). However, the credibility and the reason for this variation were not discussed. We provide n = 33 for this species and point out the need for further study of this taxon.

Even more interesting data were obtained regarding the species *Oleria didymaea* (Hewitson, 1876) and *O. onega*. We found n = 22 in the taxon identified by us as *Oleria didymaea ramona* (Haensch, 1909), whereas n= 15 was reported for taxon identified as *Oleria alexina didymaea* (Brown et al. 2004) raising the question of further study of the complex *Oleria didymaea – alexina.*

Based on chromosome numbers, we hypothesize that *Oleria onega* is a complex of at least three species with different chromosome numbers: n = 15 (our data), n = 22 and n = 30 (Brown et al. 2004). A similar conclusion can be made on the basis of molecular data that show the presence of at least four clusters of DNA barcodes in this complex (Figs 20, 21). The status of the detected chromosomal races and mitochondrial clusters could be theoretically resolved based on analysis of: (1) congruence of chromosomal and molecular characters in different sets of individuals, or (2) pattern imitating (vs not imitating) linkage of chromosomal and mitochondrial markers that are known to be unlinked (Lukhtanov at al 2015; Vishnevskaya et al. 2016, 2018; Lukhtanov 2019b). Unfortunately, the previously karyotyped samples (Brown et al. 2004) were not studied with respect to molecular markers, and vice versa, the vouchers for molecular studies were not karyotyped (de-Silva et al. 2010).

The incomplete connection between the chromosomal and molecular data (karyotypes and DNA barcodes were obtained for different sets of individuals) complicates the taxonomic interpretation of the discovered clusters. Nevertheless, we predict that in future linking karyotypes with DNA barcodes will result in a significant rearrangement of taxonomy of the genus *Oleria*.
